# Postanesthetic Effects of Isoflurane on Behavioral Phenotypes of Adult Male C57BL/6J Mice

**DOI:** 10.1371/journal.pone.0122118

**Published:** 2015-03-25

**Authors:** Kumiko Yonezaki, Kazuhiro Uchimoto, Tomoyuki Miyazaki, Ayako Asakura, Ayako Kobayashi, Kenkichi Takase, Takahisa Goto

**Affiliations:** 1 Department of Anesthesiology, Yokohama City University Graduate School of Medicine, Yokohama, Japan; 2 Department of Physiology, Yokohama City University Graduate School of Medicine, Yokohama, Japan; 3 Laboratory of Psychology, Jichi Medical University, Tochigi, Japan; Massachusetts General Hospital, UNITED STATES

## Abstract

Isoflurane was previously the major clinical anesthetic agent but is now mainly used for veterinary anesthesia. Studies have reported widespread sites of action of isoflurane, suggesting a wide array of side effects besides sedation. In the present study, we phenotyped isoflurane-treated mice to investigate the postanesthetic behavioral effects of isoflurane. We applied comprehensive behavioral test batteries comprising sensory test battery, motor test battery, anxiety test battery, depression test battery, sociability test battery, attention test battery, and learning test battery, which were started 7 days after anesthesia with 1.8% isoflurane. In addition to the control group, we included a yoked control group that was exposed to the same stress of handling as the isoflurane-treated animals before being anesthetized. Our comprehensive behavioral test batteries revealed impaired latent inhibition in the isoflurane-treated group, but the concentration of residual isoflurane in the brain was presumably negligible. The yoked control group and isoflurane-treated group exhibited higher anxiety in the elevated plus-maze test and impaired learning function in the cued fear conditioning test. No influences were observed in sensory functions, motor functions, antidepressant behaviors, and social behaviors. A number of papers have reported an effect of isoflurane on animal behaviors, but no systematic investigation has been performed. To the best of our knowledge, this study is the first to systematically investigate the general health, neurological reflexes, sensory functions, motor functions, and higher behavioral functions of mice exposed to isoflurane as adults. Our results suggest that the postanesthetic effect of isoflurane causes attention deficit in mice. Therefore, isoflurane must be used with great care in the clinical setting and veterinary anesthesia.

## Introduction

At one time, the halogenated ether isoflurane was the major anesthetic agent in clinical settings, but it is now mainly used for veterinary anesthesia. Similar to other general anesthetics, how isoflurane works in the brain remains unknown. Previous studies reported that isoflurane induces sedation by modifying the GABA_A_ receptor, two-pore potassium channel, glycine receptor, 5-HT_3_ receptor, kainate receptor, NMDA receptor, voltage-gated potassium channel, and nicotinic/muscarinic acetylcholine receptor [1–5]. These widespread sites of action suggest an array of effects of isoflurane in addition to intra-operative sedation. We previously reported a new mechanism underlying the prolonged effects of isoflurane on learning performance and the neural-based causal changes in rats; seven days after exposure to a normal anesthetic concentration of isoflurane, inhibitory avoidance learning, a kind of contextual learning, was greatly impaired, followed by impaired hippocampal long-term potentiation, increased GluA1 in the synaptoneurosomes, and reduced GluA1 ubiquitination, one of the main degradation pathways of GluA1 [6]. Our results indicate that the anesthesia changes neural plasticity, and that isoflurane-treated animals may be susceptible to external inputs for several days after administration of the anesthetic. These findings suggest that animals exposed to anesthetics may exhibit multiple potential effects on a variety of behaviors several days after exposure. Previous reports support our hypothesis that isoflurane has postanesthetic effects on behavioral phenotypes [7], though the reported effects are conflicting [8, 9]. Because the results of behavioral studies are not always consistent among research labs [10], a systematic comprehensive behavioral study of isoflurane-treated animals is needed.

In the present study, to investigate a variety of postanesthetic effects of isoflurane on behavior, we phenotyped isoflurane-treated mice by applying a comprehensive behavioral test battery. The same mice were tested using multiple behavioral assessments in each test battery, reducing the total number of mice needed to complete the study and allowing us to determine if correlative phenotypes exist across several domains of central nervous system (CNS) function. Furthermore, assessing a mouse across multiple behavioral paradigms that involve overlapping CNS circuitry will result in more confidence in any observable phenotypic differences, and more accurate demonstration on the broad range of phenotypes.

## Materials and Methods

### Ethics Statement

This study was carried out in strict accordance with the recommendations in the Guide for the Care and Use of Laboratory Animals of the Yokohama City University. The protocol was approved by the Committee on the Ethics of Animal Experiments of the Yokohama City University (Permit Number: F-A-12-022, F-A-12-033). The same mice were tested using multiple behavioral assessments in each test battery, reducing the total number of mice needed to complete the study. All efforts were made to minimize suffering.

### Animals and experimental design

Young male C57BL/6J mice (19–27 g, 6 weeks old; N = 209) were obtained from Japan SLC, Inc. (Tokyo, Japan). The animals were housed two to three per cage (square plastic cages with wire lids; width 22 cm, length 32 cm, height 11 cm) in a temperature-controlled room (23 ± 2°C) with a 12-h light-dark cycle (light period 0500–1900 h). The cage contained approximately 45 g of bedding. Standard pellet laboratory diet (Oriental Yeast Co., Tokyo, Japan) and water were provided *ad libitum*, and the cages were cleaned on a weekly basis.

Following a minimum period of 1 week after arriving at the laboratory, isoflurane exposure was performed according to the method of Uchimoto et al. [6]. To induce general anesthesia, mice were placed in a translucent plastic chamber (width 25 cm, length 17.5 cm, height 8 cm) within a thermostatic bath (34 ± 2°C). The chamber was flushed continuously with a carrier gas consisting of oxygen and nitrogen (FiO_2_ = 0.33) at 6 l/min and the mice allowed to breathe spontaneously. The rectal temperature of the animals was maintained at 36.5 ± 0.5°C. The concentration of isoflurane was maintained at 1.8% for 2 h. This concentration corresponds to 1.3 minimum alveolar concentration, because 1 minimum alveolar concentration of isoflurane is 1.4% in the adult mouse. The concentration was chosen based on data from pilot experiments in our laboratory. The carbon dioxide in the chamber was maintained at < 3 mmHg. The gases were monitored using a Capnomac ULTIMA monitor (Datex, Helsinki, Finland). In the yoked control group, one mouse at a time was placed in a plastic chamber flushed with the same carrier gas for 5 min, and then returned to its original cage. This was intended to expose the control animals to the same stress of handling as the isoflurane-treated animals before being anesthetized.

Arterial blood gases were measured during isoflurane administration. Mice were decapitated after 2 hours of exposure to 1.8% isoflurane. Then spurted fraction of blood, mainly arterial blood, was immediately collected (~200 μl). Arterial blood gases were analyzed using a Rapidlab 860 blood gas analyzer (Bayer HealthCare Diagnostics, Tarrytown, NY).

The animals were handled for a short time each day after isoflurane exposure. Control, yoked control, and the isoflurane-treated groups were housed separately. Behavioral experiments were started one week after isoflurane anesthesia. At the start of each test battery, each mouse was observed in a clean cage for a general health check (Table 1), followed by neurological screening. Posture was observed in the home cage [11–13].

**Table 1:** The items in the general health check.

Bald patch
Body size
Body weight
Crustiness around the nostrils/ eyes
Ear pinna/ Footpad color
Fur
Gait
Lesions on the feet/ tail
Posture
Scabs on the tail, rump, back
Tumor
Whisker

At the start of each test battery, each mouse was observed in a clean cage for a general health check. Posture was checked in the home cage.

### Neurological screening test

Following the general health check, each mouse was challenged to evaluate their reflex responses [11–13]. The neurological screening tests were designed to detect any gross abnormalities in physical function. The ear-twitch reflex was tested by approaching one ear from behind and touching the pinna, eliciting immediate movement of that ear. The eye-blink reflex was tested by approaching one eye with the covered end of a cotton-tipped applicator, eliciting immediate blinking by that eye. The postural reflex was tested by shaking the cage, eliciting the extension of all four legs to maintain an upright, balanced position. The righting reflex was tested by turning the mouse over onto its back, eliciting an immediate turnover response to restore the upright posture of standing on all four paws. The whisker-touch reflex was tested by lightly touching one set of vibrissae, eliciting cessation of whisker movement and causing the mouse to turn its head ipsilaterally to the vibrissae that was touched.

### Behavioral test batteries

**Fig 1:**
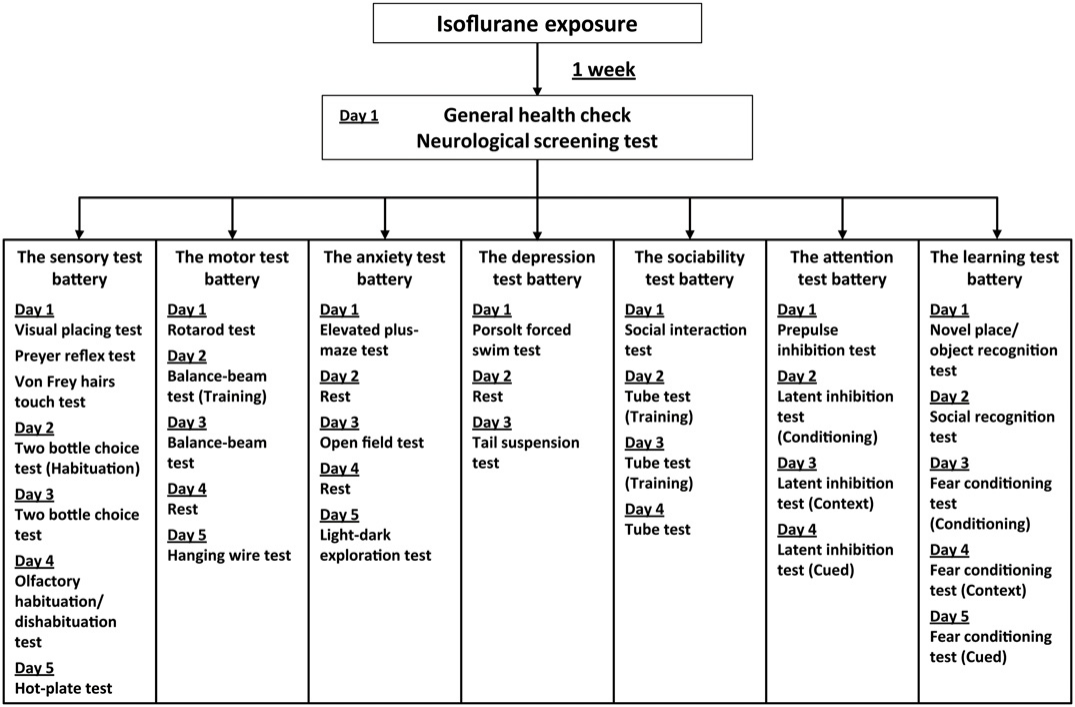
Timeline of the series of behavioral tests in each test battery. The behavioral test battery consisted of sensory test battery, motor test battery, anxiety test battery, depression test battery, sociability test battery, attention test battery, and learning test battery.

Each behavioral test battery was started when the mice were 8 weeks old (control group, N = 10; yoked control group, N = 10; isoflurane-treated group, N = 9–10), and by the end of the test battery they were 9 weeks old. Our behavioral test battery consisted of 7 test batteries as outlined in Fig. 1. To minimize potential carry-over effects between behavioral tests, the order of tests and inter-test intervals were designed based on previous reports [14, 15]. Behavioral testing, with the exception of the two bottle choice test, was performed between 0900 and 1700 h. Before performing each test, the apparatus to be used was cleaned with 70% ethyl alcohol. The experimental rooms (Room A: width 165 cm, length 165 cm, height 175 cm; Room B: width 128 cm, length 128 cm, height 195 cm; Room C: width 128 cm, length 128 cm, height 195 cm) were illuminated at 185 lux as measured at the floor of the room. The mice were habituated for 30 min before performing the behavioral tests in the room. The balance beam test, attention tests, and contextual/cued fear conditioning test were performed in Room A. The anxiety tests, depression test, novel place/object recognition test, and social recognition test were performed in Room B. The sensory tests, motor tests (except the balance beam test), and sociability tests were performed in Room C.

### Sensory test battery

#### Visual placing test

Visual function was assessed using the visual placing test [12, 13, 16]. This test involves holding the mouse by its tail approximately 30 cm above a flat table surface. As the mouse is gradually lowered to the table, it extends its forepaws for a “soft landing”. A blind mouse does not see the approaching surface and does not extend its forelimbs until its whiskers or nose touch the table. Extension of the forepaws was recorded as a yes or no response by the investigator.

#### Preyer reflex test

Auditory function was assessed by the Preyer reflex test [12, 13, 17, 18]. The Preyer reflex is a flinch response to the sound of a loud hand clap. The reflex was recorded as a yes or no response by the investigator.

#### Von Frey hairs touch test

Tactile function was assessed using the Von Frey hairs touch test [12, 19, 20]. The mouse stood on an elevated platform with wide gauge wire mesh on the surface. The Von Frey hair (2.9 *N*) was inserted through the holes in the mesh from below to poke the undersurface of a hind paw. A normal response was defined as the mouse quickly flicking its paw away from the Von Frey hair. If the mouse showed the normal responses in two out of three consecutive trials, its tactile ability was recorded as normal.

#### Two bottle choice test

Gustatory function was assessed using the two bottle choice test [12, 13]. The mouse was placed in a square aluminum cage (width 22 cm, length 32 cm, height 11 cm) and habituated for approximately 24 h. During this habituation period, the mouse was allowed to drink from two bottles containing water. After the habituation period, one of the two bottles was replaced with a bottle containing 5% sucrose and consumption monitored for 24 h as gram per gram of body weight. Standard pellet laboratory diet was provided *ad libitum* for the entire experimental period.

#### Olfactory habituation/dishabituation test

Olfaction was assessed by an olfactory habituation/dishabituation test [21]. The odorant stimuli were tap water, vanilla extract (Golden Kelly Pat. Flavor Co. Ltd., Osaka, Japan) diluted 1:100 in tap water, and bitter almond extract (Golden Kelly Pat. Flavor) diluted 1:100 in tap water. The odorant and dilution choices were based on data from pilot experiments in our laboratory. Experimenters presented stimuli by dipping a cotton-tipped applicator into the stimulus solution and then placing it through the wire lid of the cage. The applicator was stabilized 4.4 cm from the bottom of the cage. Each stimulus was presented for 3 min and then replaced by a new applicator three times in succession, for a total of nine presentations. The order of presentation was water (3x), vanilla (3x), and bitter almond (3x). An experimenter using a stopwatch recorded the cumulative time that the mouse spent sniffing the cotton-tipped applicator. Sniffing was defined as (a) tilting the head upward with the nose oriented toward and within 2 cm of the applicator, (b) rearing with the nose oriented toward and within 2 cm of the applicator, and (c) physically contacting the muzzle to the applicator if the mouth was closed. Occasional open-mouth contacts were considered to be chewing and not included in the cumulative sniff time.

#### Hot plate test

The hot plate test was performed by placing the mouse on a metal surface (19 cm in diameter, Muromachi Kikai Co., LTD, Tokyo, Japan) maintained at 54 ± 0.1°C. The hot plate was surrounded by a transparent plastic barrier 20 cm in diameter and 25 cm in height. The latency to jumping off the plate or licking a hind paw was recorded. Sixty seconds was used as a cut-off time to protect the paw against injury [12, 22].

### Motor test battery

#### Rotarod test

Motor coordination and balance were assessed using an accelerating rotarod (Muromachi Kikai Co., LTD) as described previously [15, 23]. Briefly, mice were placed on a cylinder that slowly accelerated from 4 to 40 rpm the latency to fall recorded for a maximum of 300 sec. Each mouse performed three trials.

#### Balance beam test

Motor coordination and balance were also assessed by measuring the ability of the mice to traverse a graded series of narrow beams to reach an enclosed safety platform [24]. The beams consisted of long square or round strips of wood (1 m in length) with a cross-section of 28-, 12-, or 5-mm or diameter of 28, 17, or 11 mm, respectively. The beams were placed horizontally 50 cm above the bench surface, with one end mounted on a narrow support and the other end attached to an enclosed box (20 cm square) into which the mouse could escape. Lights (1200 lux) were positioned above and to one side of the start of the beam. During training, mice were placed at the start of the 12-mm square beam and trained for 1 day (6 trials per day) to traverse the beam to the enclosed box. After the training phase, the mouse performed one trial on each of the square beams and each of the round beams, progressing from the narrowest to the widest. Mice were allowed up to 60 sec to traverse each beam. The latency to traverse each beam and the number of times the hind feet slipped off each beam were recorded for each trial.

#### Hanging wire test

Motor function was assessed using the wire hang test, which requires balance and grip strength [12, 13, 25]. A standard wire cage lid was used for this test. Masking tape placed around the perimeter of the lid prevented the mouse from walking off the edge. The test was performed by placing the mouse on the top of a wire cage lid. The investigator shook the lid lightly three times to cause the mouse to grip the wires and then turned the lid upside down. The upside-down lid was held approximately 40 cm above the cage litter, high enough to prevent the mouse from easily climbing down but not high enough to cause harm in the event of a fall. Each mouse performed two trials. The investigator used a stopwatch to time the latency in falling off the wire lid with a maximum of 60 seconds.

### Anxiety test battery

#### Elevated plus-maze test

The elevated plus-maze test was conducted as described previously [26–28]. The apparatus consisted of two open arms (29.7 x 5.4 cm^2^) and two closed arms (30 x 6 x 15 cm^3^) extending from a common central platform (6 x 6 cm^2^). A small raised lip (0.3 cm) around the perimeter of the open arms prevented the mouse from falling. The apparatus was constructed from polypropylene, with gray floor and gray walls, and elevated 40 cm above the floor. Mice were placed individually on the center square facing an open arm and allowed to freely explore the apparatus under overhead fluorescent lighting (200 lux) for 5 min. Time spent in the open arms and open and closed arm entry (all four paws in an arm) was scored by a highly trained observer using behavioral scoring software (ANY-maze, Muromachi Kikai Co., LTD).

#### Open field test

The open field test was conducted as described previously [29]. Each subject was placed in the center of a clear Plexiglas (50 x 50 x 40 cm^3^) chamber in standard room-lighting conditions (70 lux). Activity in the open field was quantitated by ANY-maze. Horizontal activity in the center (30 x 30 cm^2^) or peripheral area (distance measured by ANY-maze), total distance (cm), and time in the center or periphery were recorded in seconds. The center distance was also divided by the total distance to obtain a center distance/total distance ratio, which can be used as an index of anxiety. Data were collected during 10 min test sessions.

#### Light-dark exploration test

The light-dark exploration test was performed as described previously [26–28, 30]. The apparatus consisted of a polypropylene cage (44 x 21 x 21 cm^3^) separated into two compartments by a partition with a small aperture (12 x 5 cm^2^) at floor level. The larger compartment (28 cm long) was open-topped, transparent, and brightly illuminated by white light from a 40 W desk lamp (1000 lux). The smaller compartment (14 cm long) was close-topped and painted black. Mice were placed individually in the center of the light compartment, facing away from the partition, and allowed to freely explore the apparatus for 10 min. The number of light-dark transitions between the two compartments and the total time spent in the dark compartment were automatically recorded by ANY-maze.

### Depression test battery

#### Porsolt forced swim test

Antidepressant activity was assessed by the Porsolt forced swim test. For swim sessions, mice were placed in individual glass cylinders (24.5 cm tall, 19 cm in diameter) filled with water (23–25°C water) to a depth of 15 cm. The depth was deep enough so that mice could not support themselves by placing their paws on the base of the cylinder. The procedure was similar to that of Porsolt et al. [31]. Behavioral scoring employed a standard 6-min test duration [32]. The water was changed between subjects. All test sessions were recorded, and the duration of immobility during the last 4 min of the test period and the latency to first immobility during the 6 min test period automatically quantified by ANY-maze. A mouse was judged to be immobile when making only those movements necessary to keep its head above water.

#### Tail suspension test

Antidepressant activity was also assessed by the tail suspension test as described previously [26, 27, 33]. Mice were securely fastened to a flat metallic surface by the tip of the tail (2–3 cm) using medical adhesive tape and suspended 30 cm above the ground in a 40 cm^3^ white Plexiglas box that isolated the mouse from visual distractions but permitted observation of behavior from above. The latency to first immobility, defined as the absence of limb movement, and the time of immobility was sampled by ANY-maze.

### Sociability test battery

#### Social interaction test

Social behavior was examined using the conventional social interaction test. Each subject was placed in the center of a clear Plexiglas (30 x 36 x 17 cm^3^) chamber in standard room-lighting conditions. A naive same-sex mouse was then introduced into the test camber and allowed to explore freely for 5 min. The interaction frequency and total duration of the interaction were recorded. Social interaction comprised sniffing, grooming, exploring, following, kicking, mounting, jumping on, wrestling, and other forms of physical contact.

#### Tube test

The tube test assay was adapted from Lindzey et al. [34]. The test employed a transparent Plexiglas tube 30 cm in length with a 3 cm inside diameter, which is sufficient to permit one adult mouse to pass through without reversing direction. For training, each mouse was released at alternating ends of the tube to run through the tube, sometimes with the help of a plastic stick pushing at its back. Each animal was given eight training trials per day on two successive days. On the third day, the mice were given the test trial.

During the test trial, two mice were released simultaneously into opposite ends, and care was taken to ensure that they met in the middle of the tube. The mouse that first retreated from the tube within 2 min was designated the “loser” of that trial. In rare cases when no mouse retreated within 2 min, the tests were repeated. Between each trial, the tube was cleaned with 70% ethanol. Between the yoked control and isoflurane-treated groups, paired encounters were staged based on body weight.

### Attention test battery

#### Prepulse inhibition test

The prepulse inhibition test was performed as described by Miyakawa et al. [35] using a startle reflex measurement system (Ohara Ika Sangyo, Tokyo, Japan). Briefly, a test session began by placing a mouse in a Plexiglas cylinder, where it was left undisturbed for 5 min. The duration of white noise used as the startle stimulus was 40 msec for all trial types. The startle response was recorded for 160 msec (measuring the response every 1 msec), starting with the onset of the prepulse stimulus. The background noise level in each chamber was 70 dB. The peak startle amplitude recorded during the 160-msec sampling window was used as the dependent variable. A test session consisted of six trial types (i.e., two types for startle stimulus-only trials, and four types for prepulse inhibition trials). The intensity of the startle stimulus was 110 or 120 dB. The prepulse sound was presented 100 msec before the startle stimulus, and its intensity was 74 or 78 dB. Four combinations of prepulse and startle stimuli were employed (74–110, 78–110, 74–120, and 78–120). Six blocks of the six trial types (four trial types with the combinations of prepulse and startle stimulus and two startle stimulus-only trials) were presented in pseudorandom order, such that each trial type was presented once within a block. The average intertrial interval was 15 sec (range: 10–20 sec).

#### Latent inhibition test

This experiment was performed similar to that reported by Miyakawa et al. [35]. On the first day, each mouse was placed in a conditioning chamber (Muromachi Kikai Co., LTD). The mice were divided into two groups: pre-exposed (P) and non-pre-exposed (NP). The P group was exposed to 40 white noise tones (68 dB, 5-sec duration, 25-sec interstimulus interval), and the NP group was exposed to no stimulus during an equivalent period. Immediately after the pre-exposure period, tone—shock pairs consisting of a 5-sec tone co-terminating with a 2-sec foot shock at 0.4 mA were delivered to both groups with a 25-sec interstimulus interval. Afterward, mice remained in the chamber for 25 sec before being returned to the home cage. On day 2, the mice were placed in the conditioning chamber for 5 min to measure freezing to the context. On day 3, the mice were put in a white Plexiglas chamber scented with vanilla essence, and after 180 sec, a 180-sec tone was delivered to measure cued freezing.

### Learning test battery

#### Novel place/object recognition test

The novel place/object recognition test was performed using an open field apparatus consisting of a cubical box (30 x 36 x 17 cm^3^) made of clear Plexiglas as described by Ng et al. [36]. All behavioral events were scored and analyzed by a highly trained observer. The test consisted of three sessions with intertrial intervals of 2 min, during which mice were returned to their home cages.

During the habituation session, four different plastic objects were presented in the open field: cube (4.7 x 4.7 x 5 cm^3^); hollow cylinder (7.2 cm height, 6.5 cm diameter); solid cylinder (6 cm height, 4 cm diameter); and prism (7.3 x 7.3 x 7.2 cm^3^). Exploration of the four different plastic objects in the open field was measured every 5 min for 15 min under dim lighting (4 lux). In the place recognition session, the four objects initially placed in a square arrangement were reconfigured into a polygon-shaped pattern by moving two objects (displaced objects, DOs). The remaining two objects were left at the same location (non-displaced objects, NDOs). The time spent exploring the DOs and NDOs was recorded for 5 min and expressed as a percentage of the total time for object investigation. In the object recognition session, one of the familiar NDOs was replaced with a new object (NO) at the same location, and the two familiar DOs were removed. The time examining the NO or familiar object (FO) was recorded for 5 min and expressed as a percentage of the total time for object investigation.

#### Social recognition test

For training, mice were placed in the chamber (30 x 36 x 17 cm) with a naïve mouse for 10 min. One hour later, the mouse was returned to the chamber and exposed to the same mouse and a novel mouse for 10 min. The time spent exploring each mouse was recorded during both the training and test phases.

#### Cued/contextual fear conditioning test

This cued/contextual fear conditioning test was performed as described by Miyakawa et al. [35]. Briefly, on the first day, each mouse was placed in a conditioning chamber (Muromachi Kikai Co., LTD). The mice were habituated to the chamber for 1200 s. Immediately after exposure to the chamber, tone—shock pairs consisting of a 5-sec tone co-terminating with a 2-sec foot shock at 0.4 mA were delivered with a 25-sec interstimulus interval. Afterward, mice remained in the chamber for 25 sec before being returned to their home cage. On day 2, the mice were placed back in the conditioning chamber for 5 min to measure freezing to the context. On day 3, the mice were put in a white Plexiglas chamber scented with vanilla essence, and after 180 sec, a 180-sec tone was delivered to measure cued freezing.

### Statistical analysis

In most cases, one-way analysis of variance (ANOVA) was performed with group (control, yoked control, or isoflurane-treated) as the variable. This test was followed by post-hoc analysis with Fisher’s protected least significant difference test. For the two bottle choice test, olfactory habituation/dishabituation test, rotarod test, balance beam test (day 1), hanging wire test, prepulse inhibition test, latent inhibition test, novel place/object recognition test, social recognition test, and cued/contextual fear conditioning test, two-way ANOVA with repeated measures was performed using group and the other (bottle, odor, trial, beam, startle stimulus, object, mouse, or time) as variables. These tests were followed by post-hoc ANOVA. Chi-square analysis was used to evaluate differences due to group for the general health check, neurological screening test, visual placing test, Preyer reflex test, Von Frey hairs touch test, and tube test because the parameters were nominal scales (i.e., “normal” or “abnormal” was recorded). Differences were considered significant at p < 0.05. The data in each group for the cued/contextual fear conditioning test were reanalyzed as the data for the NP group in the latent inhibition test.

## Results

### General condition of the mice

Arterial blood gases were similar in the three groups (Table 2). The general health check indicated good general health and normal gross appearance for all mice (data not shown). Neurological reflexes were normal among the three groups. Ear twitch, eye blink, postural, righting, and whisker-orienting reflexes were similar among the three groups (data not shown).

**Table 2:** Results of arterial blood gases assessment.

Group	Control	Yoked control	Isoflurane
pH	7.34 ± 0.03	7.33 ± 0.01	7.33 ± 0.02
pCO2	35.63 ± 3.24	42.78 ± 0.74	44.58 ± 3.48

No significant differences were observed among the three groups. Values are mean ± SEM.

### Sensory functions

The results of the visual placing test, Preyer reflex, and Von Frey hairs touch test are provided in Table 3. The visual placing test indicated normal visual function in all mice, and the Von Frey hairs touch test indicated normal tactile function. The Preyer reflex was also similar among the three groups.

**Table 3:** Results of sensory tests.

Group	Control	Yoked control	Isoflurane
Visual placing test (% with normal response)	100.0 ± 0.0	100.0 ± 0.0	100.0 ± 0.0
Preyer reflex test (% with normal response)	100.0 ± 0.0	100.0 ± 0.0	100.0 ± 0.0
Von Frey hairs touch test (% with normal response)	100.0 ± 0.0	100.0 ± 0.0	100.0 ± 0.0

No significant differences were observed among the three groups. The visual placing test, Preyer reflex test, and Von Frey hairs touch test indicated normal visual, auditory, and tactile function in all mice. Values are mean ± SEM.

**Fig 2:**
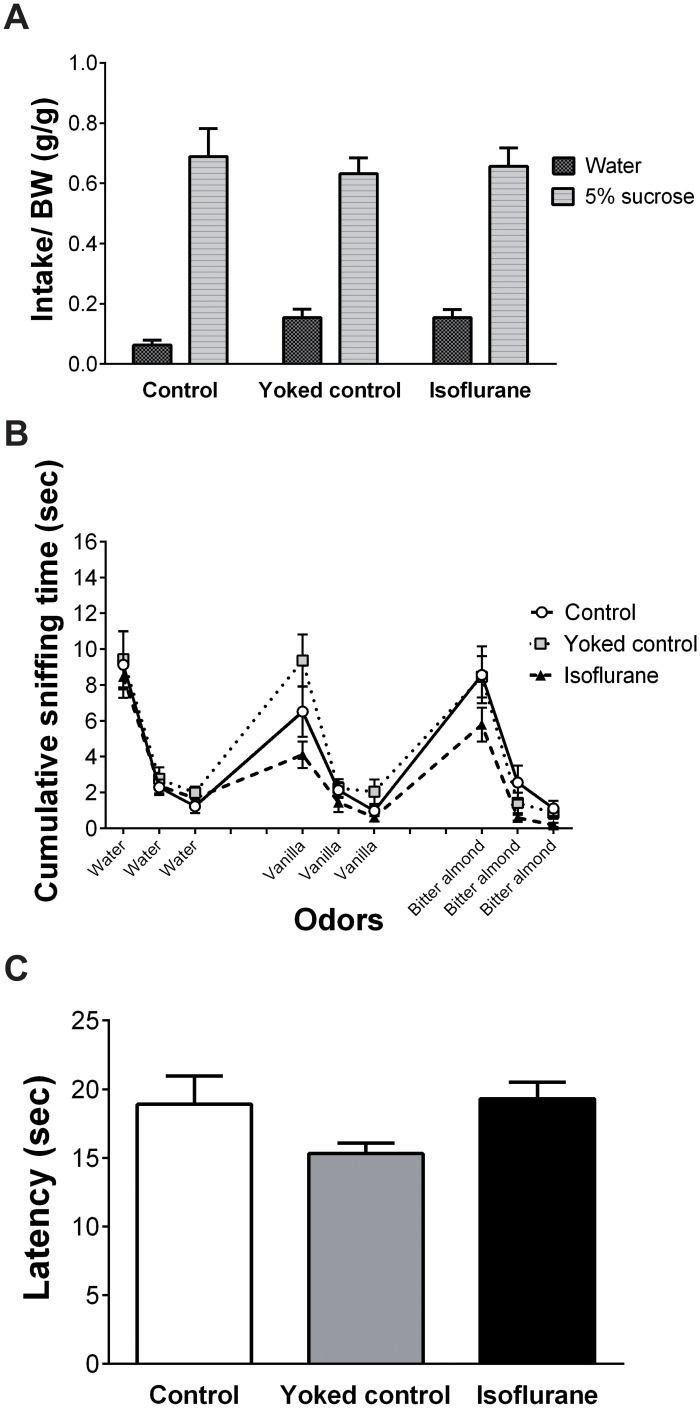
Results of the sensory tests. (A) In the two bottle choice test, no significant differences were observed among the three groups. The 5% sucrose intake was significantly greater than water intake in all groups. (B) In the olfactory habituation/dishabituation test, no significant differences were observed among the three groups. The olfactory habituation/dishabituation test indicated normal olfactory function in all groups. (C) In the hot plate test, no significant differences were observed among the three groups. Vertical lines represent the mean ± SEM.

The mean consumption of water or 5% sucrose in the two bottle choice test is shown in Fig. 2A. Two-way ANOVA with repeated measures revealed a significant main effect of bottle (i.e. water vs. sucrose; F (1, 27) = 193.45, p < 0.0001). No significant effects of group or the interaction of group × bottle were identified. The 5% sucrose intake was significantly greater than water intake in all groups.

The mean cumulative time spent on sniffing water, vanilla, or bitter almond in the olfactory habituation/dishabituation test is shown in Fig. 2B. Two-way ANOVA with repeated measures revealed a significant main effect of odor (F (8, 216) = 54.794, p < 0.0001). No significant effects of group or the interaction of group × odor were identified. Normal olfactory function was observed in all groups.

The mean latency for jumping off the plate or licking a hind paw in the hot plate test is shown in Fig. 2C. No significant differences were observed among the three groups (F (2, 27) = 2.344, p = 0.115, one-way ANOVA).

### Motor functions

#### Rotarod test

**Fig 3:**
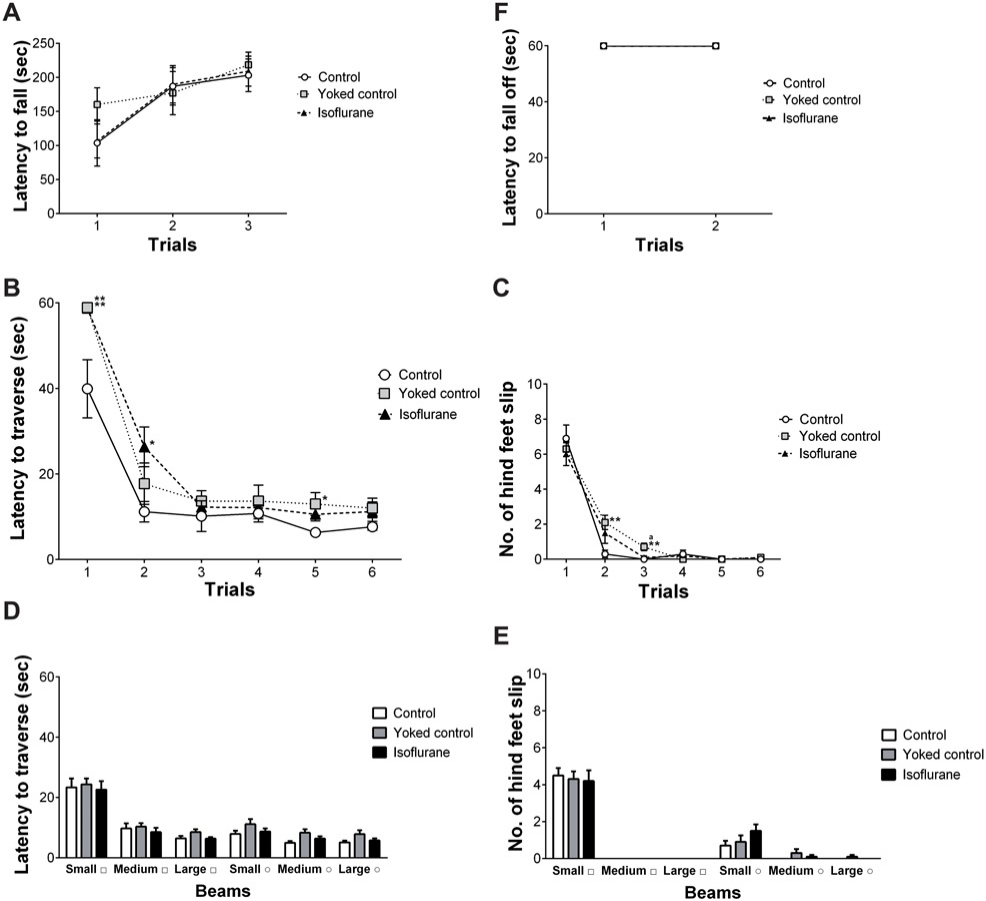
Results of the motor tests. (A) In the rotarod test, no significant differences were observed among the three groups. All groups had increased latency for falling off the rod, suggesting normal motor coordination and motor learning function. (B) In the balance beam test, the control group had decreased latency compared to the yoked control group and the isoflurane-treated group. (C) The control group also had fewer incidences of the hind feet slipping off the beam compared to the yoked control group. (D) No significant differences were observed among the three groups regarding the latency to traverse and (E) the number of times the hind feet slipped off each beam. (F) In the hanging wire test, no significant differences were observed among the three groups. The hanging wire test indicated normal balance and grip strength in all groups. * p < 0.05, ** p < 0.01 vs. control group, ^a^ p < 0.05 vs. isoflurane-treated group. Vertical lines represent the mean ± SEM.

The mean latency for falling off the rod in the rotarod test is shown in Fig. 3A. Two-way ANOVA with repeated measures revealed a significant main effect of trial (F (2, 54) = 12.123, p < 0.0001). No significant effects of group or the interaction of group × trial were identified. The rotarod test indicated normal motor coordination and motor learning function in all groups.

#### Balance beam test

The mean latency to traverse the balance beam in the training phase of the balance beam test is shown in Fig. 3B. Two-way ANOVA with repeated measures revealed a significant main effect of group (F (2, 27) = 4.067, p = 0.029), trial (F (5, 135) = 141.86, p < 0.0001), and the interaction of group × trial (F (10, 135) = 3.112, p = 0.001). Post-hoc ANOVA revealed that the control group had significantly decreased latency in the first and fifth trials compared to the yoked control group (p = 0.007 and 0.045, respectively), and that the control group had significantly decreased latency in the first and second trials compared to the isoflurane-treated group (p = 0.008 and 0.044, respectively). The mean number of times the hind feet slipped off during the training phase is shown in Fig. 3C. Two-way ANOVA with repeated measures revealed a significant main effect of trial (F (5, 135) = 193.338, p < 0.0001) and the interaction of group × trial (F (10, 135) = 2.223, p = 0.02). No significant effect of group was identified. Post-hoc ANOVA revealed that the control group had significantly fewer slips of the hind feet off the beam in the second and third trials compared to the yoked control group (p = 0.021 and 0.003, respectively), and that the isoflurane-treated group had significantly fewer slips of the hind feet off the beam in the third trial compared to the yoked control group (p = 0.013).

The mean latency to traverse each beam is shown in Fig. 3D. Two-way ANOVA with repeated measures revealed a significant main effect of beam (F (5, 135) = 71.837, p < 0.0001). No significant effects of group or the interaction of group × beam were identified. The mean number of times the hind feet slipped off each beam is shown in Fig. 3E. Two-way ANOVA with repeated measures revealed a significant main effect of beam (F (5, 135) = 150.085, p < 0.0001). No significant effects of group or the interaction of group × beam were identified.

#### Hanging wire test

The mean latency for falling off the wire in the hanging wire test is shown in Fig. 3F. Two-way ANOVA with repeated measures revealed no significant effect of group, beam, or the interaction of group × beam. The hanging wire test indicated normal balance and grip strength in all groups.

### Anxiety

**Fig 4:**
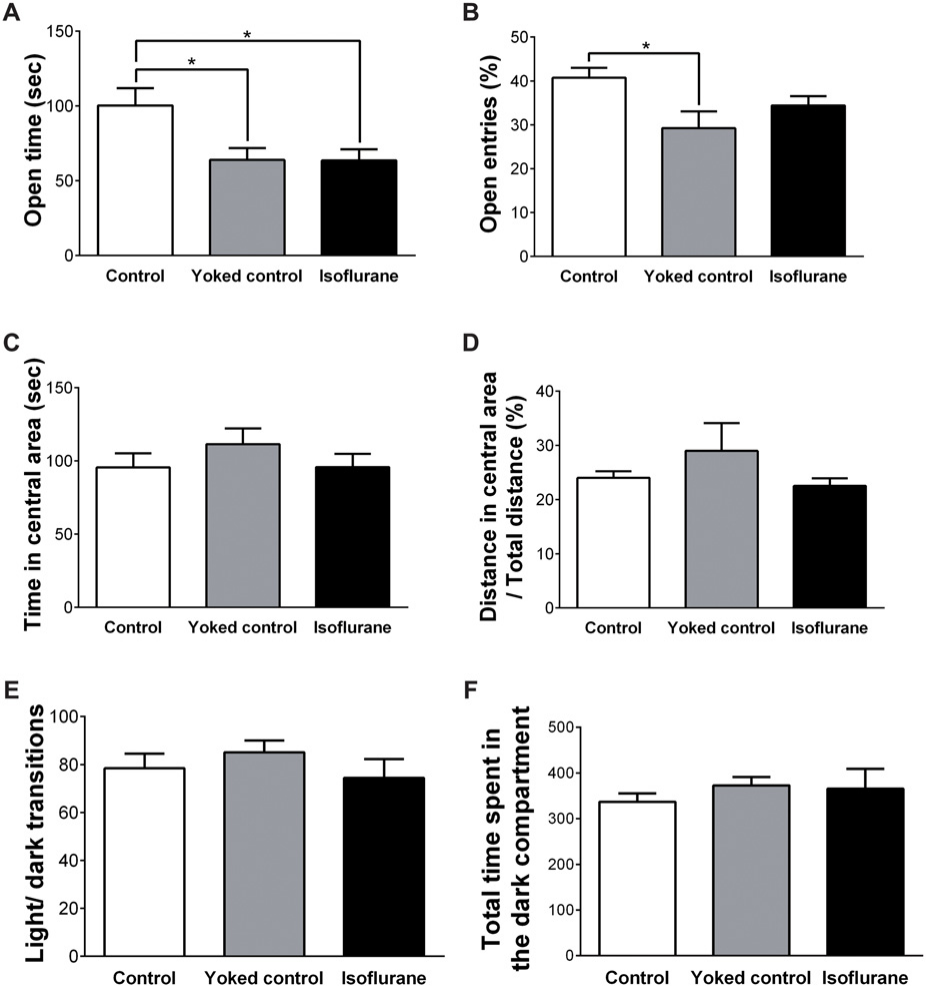
Results of the anxiety tests. (A) In the elevated plus-maze test, the mean time spent in the open arm was greater in the control group than in the yoked control group and the isoflurane-treated group. (B) The rate of open entry in the elevated plus-maze test was greater in the control group than in the yoked control group. (C) In the open field test, no significant differences were observed among the three groups regarding the time spent in the central area and (D) the distance ratio. (E) In the light-dark exploration test, no significant differences were observed among the three groups in the total number of transitions or (F) the total time spent in the dark compartment. * p < 0.05 vs. control group. Vertical lines represent the mean ± SEM.

The mean time spent in the open arm and the rate of open entry in the elevated plus-maze test are shown in Fig. 4A and 4B. For the time spent in the open arm, one-way ANOVA revealed a significant main effect of group (F (2, 26) = 5.222, p = 0.012). The mean time spent in the open arm was greater for the control group than the yoked control group (p = 0.028) and the isoflurane-treated group (p = 0.031). For the rate of open entry, one-way ANOVA revealed a significant main effect of group (F (2, 26) = 4.080, p = 0.029). The rate of open entry was greater in the control group than in the yoked control group (p = 0.025).

The mean time spent in the central area during the open field test and the distance ratio are shown in Fig. 4C and D. One-way ANOVA revealed no effect of group on the time spent in the central area (F (2, 26) = 0.853, p = 0.438) or the distance ratio (F (2, 26) = 1.098, p = 0.349).

The total number of transitions between chambers and the time spent in the dark compartment in the light-dark exploration test are shown in Fig. 4E and 4F. One-way ANOVA revealed no effect of group on the total number of transitions (F (2, 26) = 0.729, p = 0.492) or the total time spent in the dark compartment (F (2, 26) = 0.472, p = 0.629).

### Depression

**Fig 5:**
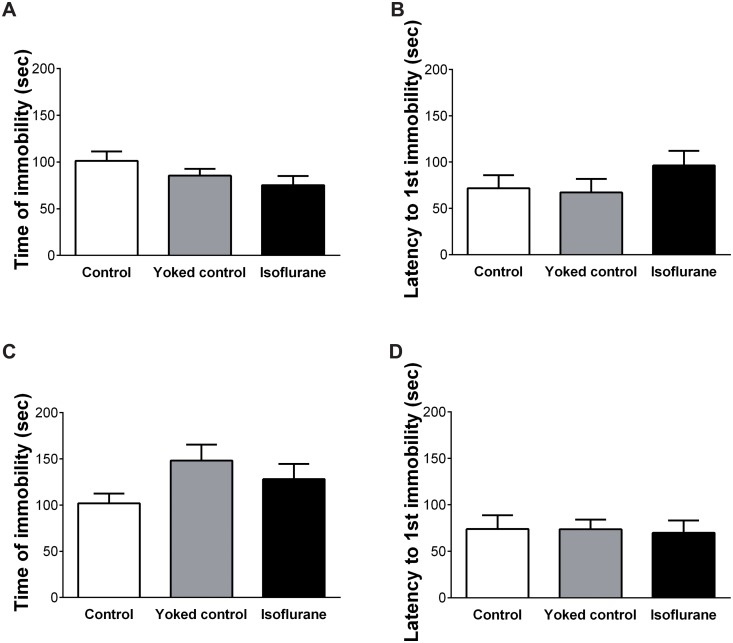
Results of the depression tests. (A) In the Porsolt forced swim test, no significant differences were observed among the three groups regarding the time of immobility or (B) the latency to first immobility. (C) In the tail suspension test, no significant differences were observed among the three groups regarding the time of immobility or (D) the latency to first immobility. Vertical lines represent the mean ± SEM.

The time of immobility during the last 4 min of the Porsolt forced swim test and the latency to first immobility during the 6 min of the test are shown in Fig. 5A and 5B. One-way ANOVA revealed no effect of group on the time of immobility (F (2, 27) = 2.046, p = 0.149) or the latency to first immobility (F (2, 27) = 1.114, p = 0.343).

The time of immobility during the last 4 min of the tail suspension test and the latency to first immobility during the 6 min of the test are shown in Fig. 5C and 5D. One-way ANOVA revealed no effect of group on the time of immobility (F (2, 27) = 2.365, p = 0.113) or the latency to first immobility (F (2, 27) = 0.031, p = 0.970).

### Sociability

**Fig 6:**
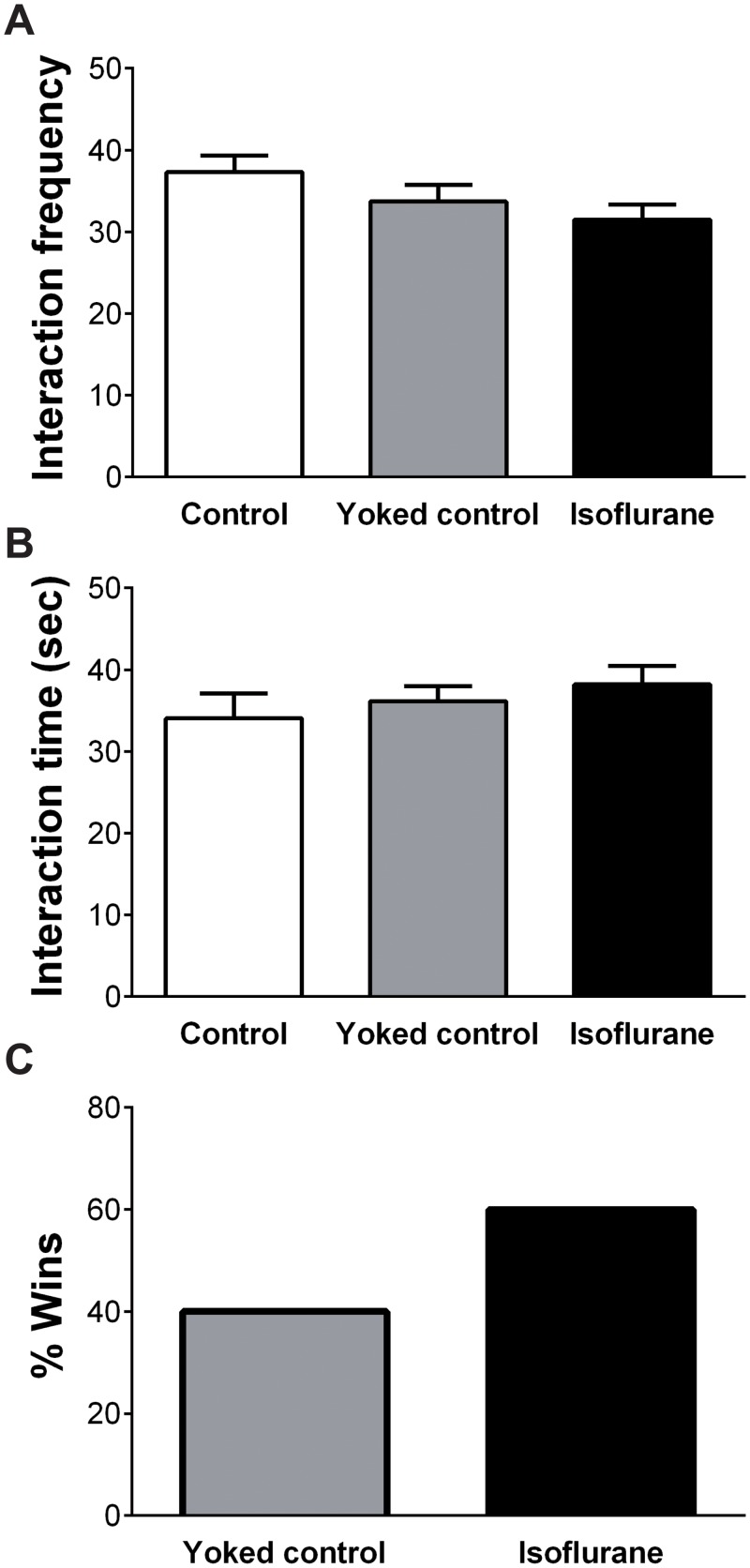
Results of the sociability tests. (A) In the social interaction test, no significant differences were observed among the three groups regarding the interaction frequency or (B) interaction time. (C) In the tube test, no significant differences were observed between the yoked control group and the isoflurane-treated group. Vertical lines represent the mean ± SEM.

The mean interaction frequency and interaction time for the social interaction test are shown in Fig. 6A and 6B. One-way ANOVA revealed no effect of group on the interaction frequency (F (2, 27) = 2.220, p = 0.128) or interaction time (F (2, 27) = 0.713, p = 0.499).

The results of tube test are shown in Fig. 6C. Chi-square analysis revealed no significant difference between the yoked control group and the isoflurane-treated group (x^2^ = 0.8, p = 0.371).

### Attention deficits

#### Prepulse inhibition test

**Fig 7:**
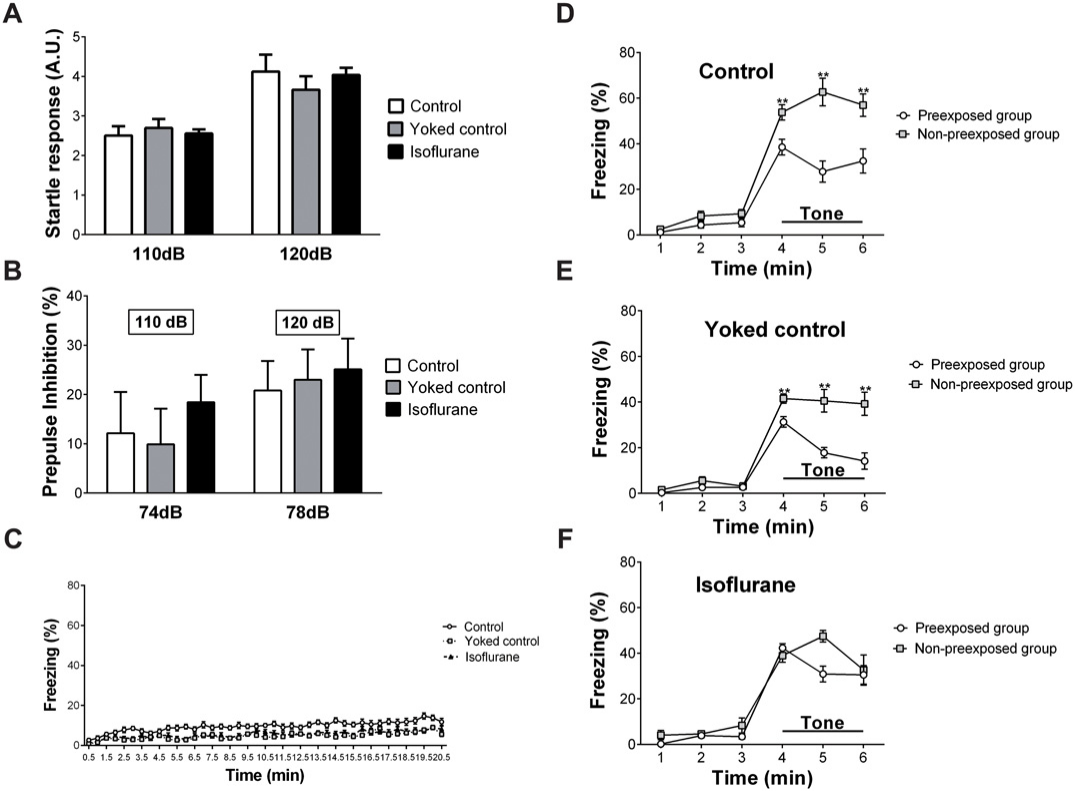
Results of the attention tests. (A) In the prepulse inhibition test, no significant differences were observed among the three groups regarding the acoustic startle response to 110 dB and 120 dB. (B) Prepulse inhibition was similar among the three groups. (C) In the latent inhibition test, the freezing level in the control group during the preshock period of the conditioning trial was slightly greater than the levels in the yoked control group and the isoflurane-treated group. (D) For the control group and (E) yoked control group, the pre-exposed group had significantly lower freezing levels during the tone-presented period than the non-pre-exposed group. (F) For the isoflurane-treated group, latent inhibition to the tone was not observed in the pre-exposed group. ** p < 0.01 vs. the corresponding pre-exposed group. Vertical lines represent the mean ± SEM.

The acoustic startle responses to 110 dB and 120 dB are shown in Fig. 7A. Two-way ANOVA with repeated measures revealed no significant main effects of group (F (2, 27) = 0.094, p = 0.910) or the interaction of group × startle stimulus (F (2, 27) = 1.707, p = 0.2). The results of the prepulse inhibition test are shown in Fig. 7B. Two-way ANOVA with repeated measures revealed no significant main effects of group (F (2, 27) = 1.536, p = 0.233), startle stimulus (F (3, 81) = 2.118, p = 0.104), or the interaction of group × startle stimulus (F (6, 81) = 0.199, p = 0.976).

#### Latent inhibition test

The freezing level in each group during the preshock period of the conditioning trial is shown in Fig. 7C. Two-way ANOVA with repeated measures revealed a significant main effect of group (F (2, 57) = 22.42, p < 0.0001) and time (F (40, 2280) = 11.075, p < 0.0001). No significant interaction of group × time was identified. The freezing level in the control group was slightly greater than the levels in the yoked control group and isoflurane-treated group.

The latent inhibition of cued fear conditioning in each group is shown in Fig. 7D-F. For the control group, two-way ANOVA with repeated measures revealed a significant main effect of group (F (1, 18) = 24.448, p < 0.0001), time (F (5, 90) = 97.415, p < 0.0001), and the interaction of group × time (F (5, 90) = 9.110, p < 0.0001). Post-hoc ANOVA revealed that the P group had significantly lower freezing levels during the tone-presented period than the NP group (p < 0.005). For the yoked control group, two-way ANOVA with repeated measures revealed a significant main effect of group (F (1, 18) = 20.516, p < 0.0001), time (F (5, 90) = 96.822, p < 0.0001), and the interaction of group × time (F (5, 90) = 11.792, p < 0.0001). Post-hoc ANOVA revealed that the P group had significantly lower freezing levels during the tone-presented period than the NP group (p < 0.004). For the isoflurane-treated group, two-way ANOVA with repeated measures revealed a significant main effect of time (F (5, 90) = 80.523, p < 0.0001) and the interaction of group × time (F (5, 90) = 2.695, p = 0.026). No significant effect of group was identified.

### Learning functions

#### Novel place/object recognition test

**Fig 8:**
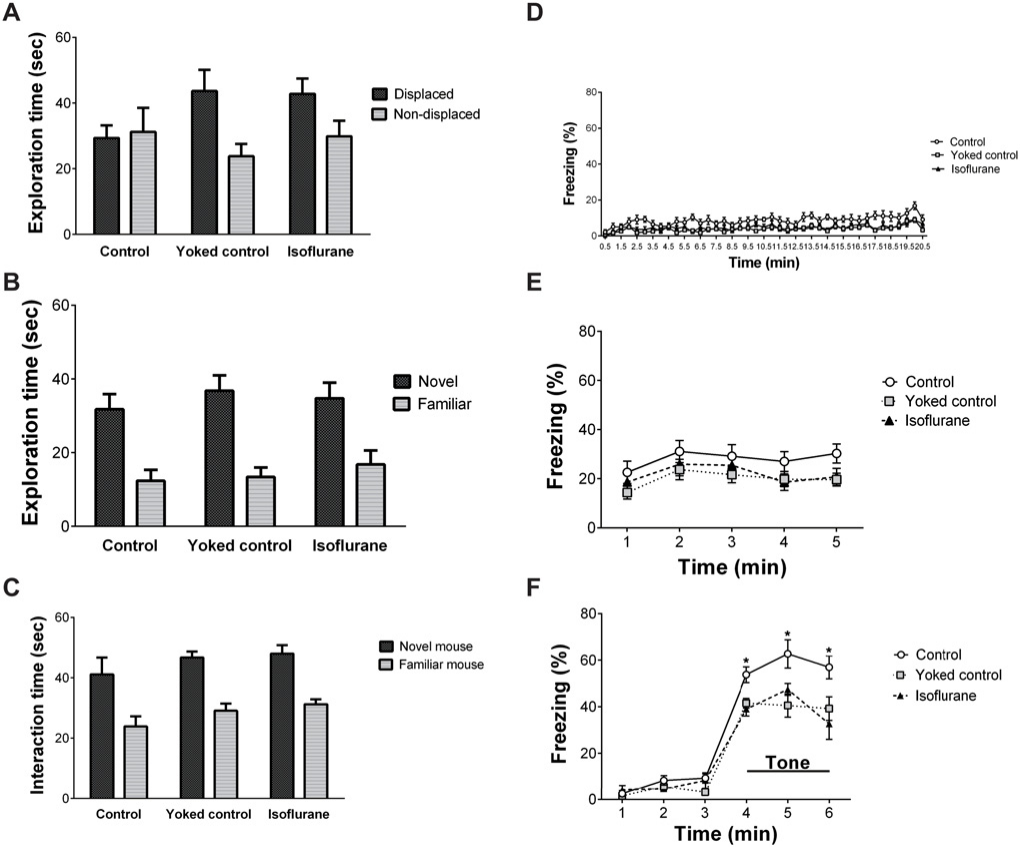
Results of the learning tests. (A) No significant differences were observed among the three groups regarding the novel place recognition test, (B) object recognition test, and (C) social recognition test. (D) In the cued/contextual fear conditioning test, the freezing level in the control group during the preshock period of the conditioning trial was slightly greater than the levels in the yoked control group and the isoflurane-treated group. (E) The freezing levels in the contextual fear conditioning test were similar among the three groups. (F) However, the control group had significantly higher freezing levels during the tone-presented period than the yoked control group and isoflurane-treated group. * p < 0.05 vs. yoked control group and isoflurane-treated group. Vertical lines represent the mean ± SEM.

The mean exploration time in the novel place recognition test is shown in Fig. 8A. Two-way ANOVA with repeated measures revealed a significant main effect of object (i.e. DO vs. NDO) (F (1, 27) = 5.369, p = 0.028). No significant effects of group or the interaction of group × object were identified. The mean exploration time in the novel object recognition test is shown in Fig. 8B. Two-way ANOVA with repeated measures revealed a significant main effect of object (i.e. NO vs. FO) (F (1, 27) = 61.447, p < 0.0001). No significant effects of group or the interaction of group × object were identified.

#### Social recognition test

The mean exploration time in the social recognition test is shown in Fig. 8C. Two-way ANOVA with repeated measures revealed a significant main effect of mouse (i.e. novel vs. familiar) (F (1, 27) = 129.246, p < 0.0001). No significant effects of group or the interaction of group × mouse were identified.

#### Cued/contextual fear conditioning test

The freezing level in each group during the preshock period of the conditioning trial is shown in Fig. 8D. Two-way ANOVA with repeated measures revealed a significant main effect of group (F (2, 27) = 7.472, p = 0.003) and time (F (40, 1080) = 5.291, p < 0.0001). No significant interaction of group × time was identified. The freezing level was slightly higher in the control group than in the yoked control group and the isoflurane-treated group. The freezing levels in the contextual fear conditioning test are shown in Fig. 8E. Two-way ANOVA with repeated measures revealed a significant main effect of time (F (4, 108) = 3.736, p = 0.007). No significant effects of group or the interaction of group × time were identified. The freezing levels in the cued fear conditioning test are shown in Fig. 8F. Two-way ANOVA with repeated measures revealed a significant main effect of group (F (2, 27) = 10.796, p < 0.0001), time (F (5, 135) = 142.608, p < 0.0001), and the interaction of group × time (F (10, 135) = 3.135, p = 0.001). Post-hoc ANOVA revealed that the control group had significantly higher freezing levels during the tone-presented period than the yoked control group and the isoflurane-treated group (p = 0.003–0.015).

## Discussion

In the present study, mice exposed to 1.8% isoflurane for 2 h, which is equivalent to normal surgical concentrations, exhibited impaired latent inhibition 7 days after anesthesia, even though the concentrations of residual isoflurane in the brain was presumably negligible at this time. In addition, the yoked control group and isoflurane-treated group exhibited higher anxiety in the elevated plus-maze test and impaired learning function in the cued fear conditioning test compared to the control group. However, no influence of anesthesia was observed on sensory function, motor function, antidepressant behavior, or social behavior. A number of papers have revealed an effect of isoflurane on animal behaviors, but until now, no systematic investigation of general health, fundamental behavior, and higher behavioral functions had been performed. To the best of our knowledge, this report is the first to systematically investigate the behavioral phenotypes of adult mice exposed to isoflurane. Our results show that anesthetics induce attention deficit in mice during the postanesthetic period.

Previous studies have reported that isoflurane strongly potentiates the activity of the GABA_A_ receptor, two-pore potassium channel, glycine receptor, 5-HT_3_ receptor, and kainate receptor, and that it greatly inhibits the activity of inwardly rectifying potassium channel and AMPA receptor. Minor inhibition of the NMDA receptor, voltage-gated potassium channel, and nicotinic/muscarinic acetylcholine receptor activity has also been observed as an effect of isoflurane [3, 4]. These results suggest that the activation/inhibition of these molecules may impair latent inhibition in isoflurane-treated mice. In fact, transgenic mice over-expressing 5-HT_3_ receptor exhibit abnormal behavior in the latent inhibition test [37]. Schmajuk et al. [38] reported that latent inhibition may be controlled by the circuit involving the entorhinal cortex, nucleus accumbens, and mesolimbic dopamine projection from the ventral tegmental area to the nucleus accumbens; thus, this circuit may be susceptible to external inputs for several postanesthetic days, not just after anesthesia.

In the elevated plus-maze, the mean time spent in the open arm was greater in the control group than in the yoked control group and isoflurane-treated group. Also, the rate of open entry was greater in the control group than in the yoked control group. These results suggest that the yoked control group and the isoflurane-treated group exhibited higher anxiety with the height (i.e., acrophobia). Furthermore, these two groups had significantly increased latency traversing the balance beam and slipped off the beam more frequently than the control group. Although the balance beam test assesses motor coordination and balance [24], anxiety caused by the height and light may influence motor performance in this test. The beam height and lighting were similar to that of the elevated plus-maze test and the light-dark exploration test. Because the yoked control group and isoflurane-treated group exhibited higher anxiety in the elevated plus-maze test, the increased latency and frequency of slipping off the beam may represent anxiety caused by the height, not impaired motor coordination. In support of this interpretation, no significant differences were observed in the rotarod test, which also assesses motor coordination and balance.

Nevertheless, why did the mice in the yoked control group and the isoflurane-treated group exhibit higher anxiety in the elevated plus-maze test? Võikar et al. [39] reported that handling manipulation and 5 minutes exposure to open field enhanced anxiety-like behavior of C57BL/6J mice in the elevated plus-maze test, whereas the same previous experiences did not affect the anxiety-like behavior of 129S2/Sv mice. This finding is in line with the reports where repeated testing enhances anxiety-like behavior in the elevated plus-maze [40–42], and further suggests that the elevated plus-maze test is the most susceptible test to previous experiences. Based on these reports, short-term placement in a translucent plastic chamber within a thermostatic bath as well as the genetic background of the mice may enhance anxiety measured by the elevated plus-maze test in the yoked control group and in the isoflurane-treated group. Furthermore, Võikar et al. [39] reported that the previous experiences did not affect the performance in the open field test and light-dark exploration test, which is consistent with our findings, and support our interpretation.

Nevertheless, the effects of previous experience on the performance in the hot plate test and forced swim test were also reported; repeated testing increased nociceptive sensitivity and decreased antidepressant activity in C57BL/6J and 129S2/Sv mice [39]. These results are inconsistent with our findings and suggest that the types of previous experiences may have differential effects on a performance in each behavioral test. In fact, the yoked control group and the isoflurane-treated group also exhibited impaired learning function in the cued fear conditioning test, although no effects of previous experience were observed in the previous report [39]. Because there are overlapping neural circuits (i.e., medial prefrontal, cingulate, and ventrolateral orbital cortices, taenia tecta, nucleus accumbens, paraventricular nucleus of the hypothalamus, medial nucleus of the amygdala and lateral septum) underlying tone-induced fear responses and elevated maze-induced anxiety states [43], short-term placement in a translucent plastic chamber within a thermostatic bath may affect these brain regions, and thus caused the behavioral changes in the elevated plus-maze test and the cued fear conditioning test.

We previously reported prolonged effects of isoflurane on contextual learning performance as assessed by the inhibitory avoidance test, as well as neural-based causal changes, in rats 7 days after exposure to isoflurane at a normal surgical concentration [6]. However, the present study did not find any change in the contextual learning performance when assessed by the contextual fear conditioning test, which requires hippocampal integrity. Two hypotheses can explain the discrepancy between our previous and present studies. First, rats may be better at hippocampal learning tasks than mice [44]. The inferior learning ability of mice could produce a floor effect; the effect of isoflurane on contextual learning function may be undetectable because the normal learning ability of mice is already minimal. Second, the difference between the shock intensities may influence the strength of the association between the context and the foot shock; a foot shock of 0.8 mA was delivered by Uchimoto et al. [6], but the present study used 0.4 mA. Because the weak shock intensity decreased the engram assessed by the contextual memory test [45], the contextual fear conditioning paradigm employed in the present study may potentiate the floor effect of isoflurane.

C57BL/6 inbred strains of mice were used for this study because the strain is widely used in knockout, transgenic, and pharmacological research and to evaluate the behaviors associated with the effects of genes and pharmacological agents using the loss-of-function and gain-of-function method. Investigation of the behavioral phenotypes of mice has contributed to our understanding of the molecular mechanisms underlying complex behaviors and establishing of the animal models originating from human CNS disorders [46–51]. Mice have long been used as animal models mimicking human behavior and are considered essential for translational research. Moreover, we designed this study to find potential behavioral effects of isoflurane. Taken together, our results indicate that isoflurane must be used cautiously in the clinical setting and veterinary anesthesia due to prolonged postanesthetic behavioral effects.

There are limitations to the present study. First, the sample size in the study may be too small to yield such conclusion. Second, 1.8% isoflurane had differential postanesthetic behavioral effects in rats [6] and mice, suggesting the differential effects in humans. Although this report is the first to systematically investigate the behavioral phenotypes of adult mice exposed to isoflurane, further studies are warranted to confirm our findings and to reveal the postanesthetic behavioral effects of isoflurane.
